# Attitude Toward Protective Behavior Engagement During COVID-19 Pandemic in Malaysia: The Role of E-government and Social Media

**DOI:** 10.3389/fpubh.2021.609716

**Published:** 2021-03-01

**Authors:** Norazryana Mat Dawi, Hamidreza Namazi, Ha Jin Hwang, Suriani Ismail, Petra Maresova, Ondrej Krejcar

**Affiliations:** ^1^Sunway University Business School, Sunway University, Selangor, Malaysia; ^2^School of Engineering, Monash University, Selangor, Malaysia; ^3^Center for Basic and Applied Research, Faculty of Informatics and Management, University of Hradec Kralove, Hradec Kralove, Czechia; ^4^Faculty of Medicine and Health Sciences, Universiti Putra Malaysia, Selangor, Malaysia; ^5^Department of Economics, Faculty of Informatics and Management, University of Hradec Kralove, Hradec Kralove, Czechia; ^6^Malaysia Japan International Institute of Technology (MJIIT), Universiti Teknologi Malaysia, Kuala Lumpur, Malaysia

**Keywords:** COVID-19, e-government, social media, protective behavior, attitude, Malaysia

## Abstract

The coronavirus disease 2019 (COVID-19) pandemic is still evolving and affecting millions of lives. E-government and social media have been used widely during this unprecedented time to spread awareness and educate the public on preventive measures. However, the extent to which the 2 digital platforms bring to improve public health awareness and prevention during a health crisis is unknown. In this study, we examined the influence of e-government and social media on the public's attitude to adopt protective behavior. For this purpose, a Web survey was conducted among 404 Malaysian residents during the Recovery Movement Control Order (RMCO) period in the country. Descriptive and multiple regression analyses were conducted using IBM SPSS software. Social media was chosen by most of the respondents (*n* = 331 or 81.9%) as the source to get information related to COVID-19. Multiple regression analysis suggests the roles of e-government and social media to be significantly related to people's attitudes to engage in protective behavior. In conclusion, during the COVID-19 outbreak, public health decision makers may use e-government and social media platforms as effective tools to improve public engagement on protective behavior. This, in turn, will help the country to contain the transmission of the virus.

## Introduction

Severe acute respiratory syndrome coronavirus 2 (SARS-CoV-2) or better known as coronavirus disease 2019 (COVID-19) first emerged in Wuhan, China, in December 2019. The disease then rapidly spread to many countries worldwide, and the World Health Organization (WHO) has declared the outbreak as a global pandemic on March 11, 2020 (1). According to the John Hopkins University COVID-19 dashboard (2), COVID-19 has infected millions of people and caused thousands of deaths worldwide. The pandemic also has pushed the healthcare system to its capacity and caused a global economic crisis.

Due to the rapid spread and absence of a vaccine or antiviral treatment, non-pharmaceutical interventions (NPIs) are the only available option to control the outbreak infection of respiratory viruses such as SARS-CoV-2 in a population (3). For instance, travel restrictions, school closures, and bans of public events have been imposed by governments across countries during the COVID-19 pandemic (4). Besides, people are advised to perform protective behavior, as recommended by WHO, such as wearing a face mask and maintaining social distance in public, regularly washing hands, staying at home, and self-isolating people with COVID-19 symptoms. Maximizing compliance with the recommended NPIs is important to delay the spread of the virus (5).

It is argued that individual behaviors in adopting protective behavior are more vital than government actions in controlling the spread of COVID-19 (6). Human behavior is the crucial element in framing the pandemic. Individuals will change their behavior spontaneously by adopting protective behavior when they perceive a high risk of infection and understand the severity of the disease. This behavior in return will help to reduce the transmission of the virus (7). However, a key problem is how the population perceives the risk to influence its engagement in protective behavior. According to the Theory of Reasoned Action (TRA), one of the factors that contribute to intended behavior is attitude (8). Attitude has long been recognized as a factor that leads people to perform a particular behavior (9). Attitude refers to the evaluative outcome of performing a specific behavior (8). If individuals have positive attitudes toward the suggested behavior, they are more likely to perform the behavior. Therefore, understanding individual attitudes toward protective behavior adoption is important to assess their adoption behavior and, consequently, enable public health authorities to assess the effectiveness of NPIs during the COVID-19 pandemic.

Risk communication strategies conducted by the government are highly effective and less costly NPIs during the COVID-19 outbreak (10). During a pandemic, people have a perception that the government has to play an active role to protect and ensure public safety (11). People will be motivated to engage in protective behavior when the government shows its determination to control the spread of the virus. Due to the restricted movement and social distancing, the COVID-19 pandemic has led to the urgency for e-government services. The United Nations stated that during the COVID-19 outbreak, governments around the world started using digital platforms such as portals, social media, and mobile applications to provide information related to COVID-19 to the public (12). Among the basic information given are travel restrictions, guidance on preventive measures, and governmental responses. The enhancement in e-governance during the COVID-19 pandemic has helped governments to combat the effects related to the pandemic (13). A study conducted in China reported that the use of social media by the Chinese government to provide the latest news in handling the COVID-19 crisis had positively affected public engagement (14).

In the past few years, there is an increase in governmental efforts to improve public communications using social media (15). With more than 3 billion users worldwide (16), social media could be the best platform to increase people's awareness and adherence to the recommended protective measures. Functions such as banners and pop-ups in social media are beneficial to alert users on new updates and to give reminders on protective behaviors such as social distancing and handwashing (17). Furthermore, the ability of social media to share real-time information will help to detect and combat infectious diseases. According to the social media analytics platform Sprinklr, there were about 19 million mentions of coronavirus across social media in 24 h after WHO announced COVID-19 as a pandemic (18). It shows the important role played by social media in disseminating information during the health outbreak.

E-government and social media are important platforms to supply information related to COVID-19 and to educate people on protective behavior. As people limit their physical interaction, stay more at home, and have more online time during the pandemic, their attitude toward protective behavior adoption could be influenced by the exposure to e-government and social media. However, there is still an unclear understanding of the roles played by these 2 variables in health promotion during a pandemic. In an investigation by Yasir et al. (11), results affirmed that there are positive relationships between the role of e-government and word of mouth with social presence during the COVID-19 outbreak. Further, Ahmad et al. (19) figured out that the government's guidelines on epidemic prevention influence individuals' intention to adopt COVID-19 prevention methods, while Nazir et al. (20) found that social media indirectly influences preventive behavior through awareness and information exchange. To the best of our knowledge, no study has been conducted to test the associations between e-government and social media with the attitude toward protective behavior during the COVID-19 pandemic. Therefore, empirical research is needed to test the relationships.

Malaysia is among the countries that were hit by the virus at the early stage of the outbreak due to its close location to China. The country detected the first case of COVID-19 on January 24, 2020, involving 3 tourists from Wuhan, China (21). To contain the spread of the virus, the Malaysian government has taken proactive actions such as health screening at all points of entry, compulsory quarantine for international travelers, increasing the number of hospitals to treat COVID-19 cases, setting up COVID-19 Fund, and implementation of Movement Control Order (MCO) (22).

Concerned about the importance of effective risk communication during the pandemic, the government of Malaysia relies heavily on e-government and social media to provide sufficient and up-to-date information to the public. Effective risk communication during a health crisis helps not only to relieve panic among society but also to promote adoption of protective measures (23). Malaysia residents can get information and updates on COVID-19 through the Official Portal of the Ministry of Health Malaysia, special Facebook pages called the Crisis Preparedness and Response Center (CRPC) and Kementerian Kesihatan Malaysia (KKM), and also Telegram channel of CPRC KKM (24). Daily press conference by the Director-General of Health provides consistent updates of COVID-19, which was broadcast not only through television but also online streaming on Facebook. In April 2020, a mobile application called MySejahtera was launched by the government to help in managing the outbreak in the country. This application assists users to monitor their health progress and register their check-in locations, helping the authorities to gather early information and provide effective and fast responses to control the spread of COVID-19.

**Figure 1:**
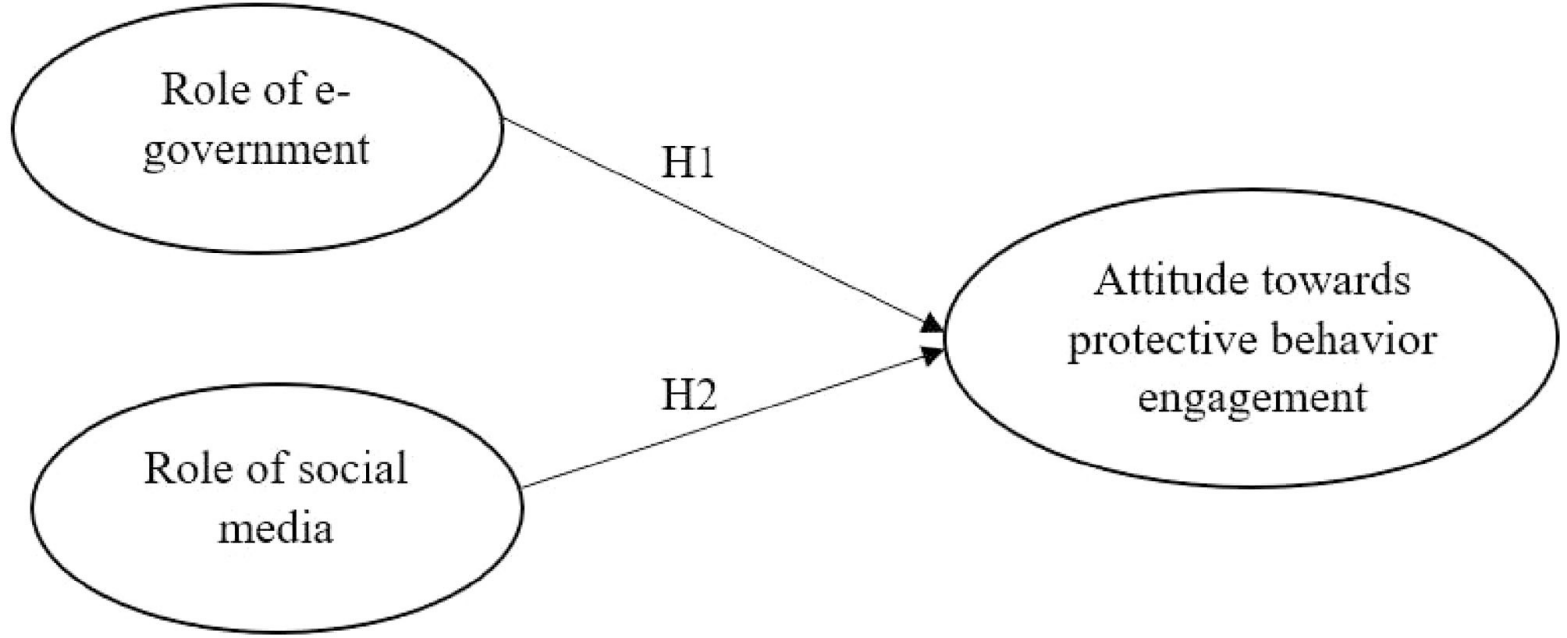
Research model.

In June 2020, the Malaysian government reopened the economy and lifted some restrictions in phases as the number of cases had been declining (25). As of the date of writing, September 10, 2020, Malaysia's COVID-19 data show that there have been 9,628 total cases, 9,167 fully recovered, and 128 deaths. Even though Malaysia is facing downtrends of new and active COVID-19 cases, the condition is still concerning, as currently, there is still no vaccine available to fight the disease (26). Improving public attitudes toward adopting protective behavior is therefore of particular importance in this country. The purpose of this study was to explore the roles played by e-government and social media on Malaysian residents' attitudes to engage in protective behavior (Figure 1). It is deemed important to test the research framework in the context of Malaysia, as e-government and social media are used widely by health authorities for public health communications during the COVID-19 outbreak. It is argued that the successful interventions by the Malaysian government had improved the compliance and cooperation of the public to combat the disease (27). Assessing this phenomenon would be helpful for the public health decision makers to design preventive and mitigation strategies as the pandemic evolves. Given the above literature, we hypothesized the following:

H1: The role of e-government is positively related to attitude toward protective behavior engagement.
H2: The role of social media is positively related to attitude toward protective behavior engagement.

## Methodology

Data for the study were collected using convenience and snowball sampling techniques. A Web-based survey was used to collect the data. Invitations to participate in the study were sent to Malaysian residents using multiple platforms such as social media, e-mail, and Messenger during the COVID-19 Recovery Movement Control Order (RMCO) period in the country. The questionnaire was prepared in English language, and a total of 404 participants have taken part in the survey. Most of the participants were Malaysians, and we also included non-Malaysians who were residing in the country. IBM SPSS Version 23 was used to conduct data cleaning, descriptive analyses, and multiple regression analysis. All the questions were adapted from different published literature and were modified to suit health promotion context during the COVID-19 outbreak. Specifically, 8 items for the role of the e-government were adapted from Parrey et al. (28) and Park and Lee (29), 9 items for the role of social media were adapted from Parrey et al. (28) and Karasneh et al. (30), and 9 items for the attitude toward protective behavior engagement were adopted from Ajzen and Fishbein (31) and World Health Organization (32). The instruments were given to 4 lecturers in health science and information systems to confirm that the concepts are appropriate for the health promotion context. Ethical approval was obtained from the Monash University Human Research Ethics Committee (Project ID: 24906).

## Results

### Profile of Respondents

Table 1 presents the demographic characteristics of the 404 participants. There were 263 females (65.1%) and 141 males (34.9%) who were included in this study. The majority of the respondents (336) were young adults aged between 18 and 24 years, which accounted for 83.2% of the total respondents. In terms of nationality, 83.9% (*n* = 339) of the sample were Malaysians, while the remaining 16.1% (*n* = 65) were non-Malaysians. The distribution by academic qualifications indicated that most of them (*n* = 207, 58.7%) held a bachelor's degree.

**Table 1:** Participants' demographic profiles.

**Variables**	**Frequency (*n*)**	**Percentage (%)**
**Gender**		
Male	141	34.9
Female	263	65.1
**Age**		
18–24	336	83.2
25–34	29	7.2
35–44	24	5.9
45–54	12	3.0
55–64	3	0.7
**Academic qualifications**		
PhD	11	2.7
Master's degree	19	4.7
Bachelor's degree	207	51.2
Diploma	32	7.9
A-Level	121	30.0
Secondary/primary school	14	3.5
**Nationality**		
Malaysian	339	83.9
Non-Malaysian	65	16.1

Participants were asked about the sources they used to get information related to COVID-19. This was a multiple-response question where participants can choose more than one answer. As shown in Table 2, 81.9% (*n* = 331) of the participants used social media to acquire COVID-19 information and 47.5% (*n* = 192) chose broadcast and print media (television, radio, and newspaper). The Ministry of Health Malaysia's website was chosen by 44.1% (*n* = 176) of the respondents, while 38.2% (*n* = 156) of them chose family/friends to get information related to COVID-19. Lastly, 21% (*n* = 85) of the respondents used the WHO website to obtain COVID-19 information.

**Table 2:** Source to get information related to COVID-19.

**Source of information**	**Frequency (*n*)**	**Percentage (%)**
Ministry of health Malaysia website	176	43.6
Social media	331	81.9
Television/radio/newspaper	192	47.5
WHO website	85	21.0
Family/friends	156	38.6

*COVID-19, coronavirus disease 2019*.

Table 3 shows the results of the question on the source to get information related to COVID-19 compared against gender. The majority of the participants who have chosen social media as the source to get information were female (*n* = 214, 64.7%), and 35.3% (*n* = 117) were male. There is a significant difference between the gender that chose family/friends as the source to obtain information related to COVID-19; 111 (71.2%) were female, and only 45 (28.8%) were male. Almost similar distribution can be seen between gender on the options of the Ministry of Health Malaysia Website (42.6% or *n* = 75 male, 57.47% or *n* = 101 female) and WHO website (44.7% or *n* = 38 male, 55.3% or *n* = 47 female).

**Table 3:** Source to get information related to COVID-19 based on gender.

**Variable**	**Gender**				**Total**	
	**Male**		**Female**			
	***n***	**%**	***n***	**%**	***n***	**%**
Ministry of health Malaysia website	75	42.6	101	57.4	176	100
Social media	117	35.3	214	64.7	331	100
Television/radio/newspaper	74	38.5	118	61.5	192	100
WHO website	38	44.7	47	55.3	85	100
Family/friends	45	28.8	111	71.2	156	100

*COVID-19, coronavirus disease 2019*.

Table 4 highlights the distribution of answers on the question of the source to get information related to COVID-19 compared against the age group. Most participants who reported using social media as the source to get COVID-19 information were 18–24 years of age (*n* = 283, 85.5%) followed by participants aged 25–34 years (*n* = 24, 7.3%); 15 (4.5%) were 35–44 years of age, and 8 (2.4%) were aged 45–54 years. In comparison with other sources, most participants (*n* = 18, 10.2%) in the age group 35–44 years chose the Ministry of Health website to get information related to COVID 19. Additionally, 2 participants (1.0%) in the age group 55–64 years have chosen television/radio/newspaper as their source to obtain information related to COVID-19.

**Table 4:** Source to get information related to COVID-19 based on age group.

**Variable**	**Age**										**Total**	
	**18–24**		**25–34**		**35–44**		**45–54**		**55–64**			
	***n***	**%**	***n***	**%**	***n***	**%**	***n***	**%**	***n***	**%**	***n***	**%**
Ministry of health Malaysia website	130	73.9	20	11.4	18	10.2	8	4.5	0	0.0	176	100
Social media	283	85.5	24	7.3	15	4.5	8	2.4	1	0.3	331	100
Television/radio/newspaper	162	84.4	13	6.8	12	6.3	3	1.6	2	1.0	192	100
WHO website	72	84.7	6	7.1	5	5.9	2	2.4	0	0.0	85	100
Family/friends	142	91	7	4.5	4	2.6	3	1.9	0	0.0	156	100

*COVID-19, coronavirus disease 2019*.

### Reliability and Validity

The validity of the instrument was assessed by conducting factor analysis, and the internal consistency reliability was checked using Cronbach's alpha coefficient.

To validate whether respondents perceived the role of e-government and the role of social media to be distinct, a factor analysis with varimax orthogonal rotation was conducted. Bartlett's test of sphericity yielded a value of 3,553.934 and an associated level of significance smaller than 0.001, while the Kaiser–Meyer–Olkin (KMO) measure of sampling adequacy was 0.908. These indicate that there is a sufficient intercorrelation among the variables. KMO value >0.90 is considered “marvelous” (33). As can be seen in Table 5, the results of factor analysis validate that the two constructs are unidimensional and factorially distinct and that the items clustered on the constructs that they were supposed to represent.

**Table 5:** Factor analysis results.

	**Factor**	
**Items**	**Role of e-government**	**Role of social media**
The quality of information (e.g., daily update on cases, preventive methods) related to COVID-19 that the government provided through social media (e.g., Facebook, Twitter, Telegram) during the COVID-19 outbreak is satisfactory	0.677	
The online services I received from public servants (e.g., police, healthcare provider, immigration office, tax office) during the COVID-19 outbreak are satisfactory	0.631	
I feel the government is committed in curbing the COVID-19 outbreak by promoting a healthy lifestyle to the people through social media	0.691	
I feel the policies and regulations that the government is imposing during the COVID-19 outbreak are favorable for the people	0.702	
I consider the government as a trustworthy source for providing COVID-19 information	0.809	
I trust COVID-19 information acquired from the government is competent to help its citizens	0.829	
I trust the government in providing me with reliable information for protecting my safety from COVID-19	0.823	
I depend on the government to obtain COVID-19 information I need	0.693	
I consider opinion from social media while selecting information related to COVID-19		0.592
I feel social media is a good source to get information on COVID-19 preventive measures		0.751
I can change my opinion about COVID-19 based on updates reported in social media		0.719
Social media plays an important role in educating me about the procedures to follow in the event of COVID-19 outbreak		0.825
Social media plays an important role in increasing my knowledge of general preventive behaviors to control the infection		0.817
Social media plays an important role in spreading awareness of COVID-19 in the community		0.702
Social media plays an important role in educating people on how to protect others if they are ill		0.761
Social media plays an important role in decreasing fear, anxiety, and confusion about COVID-19 among people		0.619
I trust in what is posted on social media related to COVID-19		0.695

The reliability of all scales was assessed based on Cronbach's alpha coefficient value. As shown in Table 6, Cronbach's alpha for the role of e-government, the role of social media, and attitude toward protective behavior were 0.884, 0.890, and 0.911, respectively, which were all above the threshold level of 0.7 (34). On average, respondents have a high level of agreement for all 3 constructs, with mean values higher than 3.

**Table 6:** Descriptive statistics and scale reliability for each construct.

**Construct**	**Items**	**Mean**	**Standard deviation**	**Cronbach's α**
Role of e-government	8	4.112	0.580	0.884
Role of social media	9	3.836	0.616	0.890
Attitude	9	4.710	0.385	0.911

The assumptions of multiple linear regression were examined. Multicollinearity was checked using intercorrelations between the predictor variables. As shown in Table 7, the role of e-government and the role of social media were significantly (*p* < 0.01) correlated with values below the threshold of 0.7. In addition, the values of variance inflation factor (VIF) are <10 (both role of e-government and role of social media have similar values, tolerance = 0.826, VIF = 1.211), which indicate no violation of the regression assumptions of multicollinearity. The assumption of independent errors was met with the value of Durbin–Watson = 1.964. Lastly, an examination of the normal *P-P* plot of regression standardized residual of attitude toward protective behavior found that it looked normally distributed.

**Table 7:** Intercorrelations of the main variables.

**Variable**	**Attitude**	**Role of e-government**	**Role of social media**
Attitude	1.000		
Role of e-government	0.362^*^	1.000	
Role of social media	0.258^*^	0.417^*^	1.000

* *p < 0.01*.

### Hypothesis Testing

Multiple regression analysis was carried out to test the relationship between the role of e-government and the role of social media toward attitude toward protective behavior engagement. The results are presented in Table 8. The model was significant (*p* < 0.01) with *F*-value of 31.929. The coefficient of determination (*R*^2^) value is 0.145, indicating that 14.5% variance in attitude toward protective behavior engagement is influenced by the role of e-government and the role of social media. The role of e-government (β = 0.205, *p* < 0.01) and the role of social media (β = 0.081, *p* < 0.05) were statistically significant predictors of attitude toward protective behavior engagement against COVID-19, supporting H1 and H2. A closer assessment of the β value showed that the role of e-government provides a greater influence on attitude compared to the role of social media.

**Table 8:** Result of multiple regression analysis.

**Model**	**Unstandardized coefficients**		***t*-test**	***P*-value**	***R***	***R*^**2**^**	***F*-test**	***P*-value**
	**β**	**SE**						
Constant	3.558	0.147	24.177	0.000	0.381	0.145	31.929	0.000
Role of e- government	0.205	0.035	5.874	0.000	N/A	N/A	N/A	N/A
Role of social media	0.081	0.033	2.473	0.014	N/A	N/A	N/A	N/A

## Discussion

During a pandemic such as COVID-19, an individual's adherence to health recommendations could be influenced by a variety of variables. From the results, we conclude that e-government and social media played significant roles in affecting Malaysian residents' attitudes toward protective behavior engagement.

Social media was chosen by most of the respondents as the source to obtain information related to COVID-19 followed by mass media. This was consistent with a study conducted by Mubeen et al. (35) to measure public awareness on COVID-19 transmission where social media was the most sought-after source of coronavirus followed by television. Another recently published study among nurses and physicians also found that social media was used to obtain information related to COVID-19 (36). This finding gives insight into the importance of public authorities to ensure that information related to the pandemic shared in social media is trustworthy and accurate.

The results of multiple regression analysis showed that e-government and social media positively predicted people's attitudes toward protective behavior engagement. This suggests the importance of continuing to provide credible information to the public through digital platforms. Our result was supported by Yasir et al. (11), who concluded that e-government gives a strong effect on the public's attitude toward quarantine during the COVID-19 outbreak in China. Government involvement in providing emergency information during an infectious disease outbreak contributes to protective behavior engagement (37, 38). People were more likely to follow public health recommendations if they were made aware of the reality of the crisis and how the government handles it (39). In addition, Park and Lee (29) figured out that e-government application was accepted by the public as a platform for public health risk communication.

Even though social media have long been acknowledged as the platform to spread false health information (40) and spreading panic during a health crisis (41), this study found that during the COVID-19 outbreak, social media is useful to influence the public to engage in protective behavior. Of note, the Malaysian government has taken serious action on misleading information related to COVID-19 posted on social media. A study by Azizan et al. (42) demonstrated that Malaysians expressed their solidarity and empowerment to fight the crisis on Facebook during the outbreak. This implies the positive effects given by social media to Malaysians in handling the unprecedented event. Our finding is consistent with that of Lin et al. (43) where they found a significant relationship between social media exposure and intention to implement preventive behavior. Indeed, social media has been regarded as an effective platform to communicate health information to the public (44).

This research has several limitations and future directions. Firstly, the sample size of this study is relatively small to generalize the population of Malaysian residents. A bigger study size should be carried out to get a better understanding of the situation. Secondly, the self-reported answers may be subject to bias, and respondents might have given socially desirable responses. Future studies may include a data collection method that does not require respondents to provide answers such as observation. Third, a previous study figured out that people's reactions to protective measures are varied across countries depending on their perceptions of their authorities (45). A comparative study between countries is recommended to extend the applicability of the current findings. Despite these limitations, the findings contribute to the understanding of the public attitude to engage in protective behavior in response to the COVID-19 outbreak in Malaysia.

## Conclusion

As the COVID-19 outbreak continues, it is crucial to take effective measures to fight the pandemic effectively. Adherence to recommended protective behaviors such as washing hands regularly, wearing face masks, and physical distancing is important to ensure public health. A strong public attitude to support the preventive measures will allow minimizing the transmission of COVID-19. The findings of this study will benefit future planning if another outbreak wave occurs. E-government and social media have been proved to influence people's attitudes to engage in protective behavior. Thus, the authority should focus on improving digital services to ensure effective risk communication and information flow to the public during a pandemic and at the same time help to mitigate the spread of the disease.

## Data Availability Statement

The raw data supporting the conclusions of this article will be made available by the authors, without undue reservation.

## Ethics Statement

The studies involving human participants were reviewed and approved by Monash University Human Research Ethics Committee. The patients/participants provided their written informed consent to participate in this study.

## Author Contributions

NM: conceptualized the idea, data collection, data analysis, and writing the original manuscript. HN: contributed to data collection and data analysis. HH: conceptualization and data collection. SI: conceptualization and editing original draft. PM: funding and edited the original draft. OK: funding and supervised this project. All authors contributed to the article and approved the submitted version.

## Conflict of Interest

The authors declare that the research was conducted in the absence of any commercial or financial relationships that could be construed as a potential conflict of interest.
